# A cross-sectional analysis of the association between self-employment, racial and ethnic minority status, sex and cardiovascular disease risk factors among a nationally representative sample

**DOI:** 10.1186/s12889-025-22955-2

**Published:** 2025-05-30

**Authors:** Kimberly Narain, Daniela Markovic, José J. Escarce

**Affiliations:** 1 Division of General Internal Medicine and Health Services Research, Department of Medicine, David Geffen School of Medicine, University of California, Los Angeles, 1100 Glendon Ave, Suite 850 & 900, Los Angeles, CA 90024 USA; 2 Iris Cantor UCLA Women’s Health Center, 100 Medical Plaza Driveway Suite 250 & 290, Los Angeles, CA 90095 USA; 3 Department of Health Policy and Management, UCLA Fielding School of Public Health, 650 Charles E. Young Dr. South, 16–035 Center for Health Sciences, Los Angeles, CA 90095−1772 USA

**Keywords:** Cardiovascular disease, Work, Public health, Sex

## Abstract

**Background:**

There is a body of evidence that suggest risk factors for cardiovascular disease (CVD) may be linked with self-employment status. Work context varies across race, ethnicity and sex. The objective is to examine the association of self-employment status and CVD risk factors across racial and ethnic minority status as well as sex.

**Methods:**

For this observational study, National Health and Nutrition Examination Survey (NHANES) data (1999–2018), a cross-sectional study design, and stratified logistic regression models were used to explore the association between self-employment status (a dichotomous variable) and CVD risk factors (dichotomized measures of elevated cholesterol, hypertension, glucose intolerance, obesity, poor diet, physical inactivity, smoking, binge drinking, sub-optimal sleep duration and poor mental health) across combined racial and ethnic minority status and sex groups, among working, non-pregnant adults(ages 30–62). Statistical models controlled for age, education, marital status, household poverty-to-income ratio, and the number of months working at current job. The coefficient estimates were expressed as predictive margins.

**Results:**

The study sample was comprised of 19,395 working adults. Among non-minority women, self-employment was negatively associated with obesity (% diff = -7.4%; *p* = 0.008), physical inactivity % diff = -7.0%; *p* = 0.017), and poor sleep duration (% diff = -9.4%; *p* = 0.004). Among minority women, self-employment was negatively associated with poor diet (% diff = -6.7%; *p* = 0.024), physical inactivity (% diff =-7.3%; *p* = 0.013) and poor sleep duration (% diff = -8.1%; *p* = 0.017). Among non-minority men, self-employment was negatively associated with poor diet (% diff = -6.5%; *p* = 0.008) and hypertension (% diff = -5.7%; *p* = 0.013).

**Conclusions:**

This study suggests that there may be a relationship between work context and CVD risk factors that varies across race, ethnicity and sex; however, further research is needed to characterize this relationship. Specifically, exploring how autonomy, flexibility, social support and discrimination exposure varies across self-employment status in diverse demographic groups may be important for illuminating the relationship between work and cardiovascular health.

## Introduction

Cardiovascular disease is the leading cause of death among Americans. In 2021, roughly 931,578 individuals lost their lives due to cardiovascular disease [1]. Key drivers underlying the high prevalence of cardiovascular disease in the United States include poor dietary quality, hypertension, excess body weight, insulin resistance, high cholesterol and inadequate sleep [2, 3, 4]. There is a body of evidence that suggest risk factors for cardiovascular disease may be linked with the structure of employment. Early landmark studies linking socioeconomic status and health, such as the Whitehall Studies, focused on employment type and found that those in executive positions had better health outcomes than those in lower position jobs (clerical and administrative) [5]. More recent studies have shed light on the employment characteristics underlying these ground-breaking findings. For example, the health benefits of occupation are partially mediated by job control [6, 7, 8]. Specifically, jobs with higher psychological demands and less autonomy (high strain jobs) have been consistently linked with hypertension and cardiovascular disease [11, 12]. Beyond the specific work tasks, characteristics of the work contract are also associated with health. Effort-reward imbalance which refers to an imbalance between effort spent at work (time pressure and demands) and rewards (esteem; promotion; job security) has also been linked with cardiovascular disease [11, 12, 13].

People of color and women are often exposed to less healthy work environments. Specifically, people of color are over-represented in high strain occupations [9, 10, 11] and experience higher levels of effort-reward imbalance, even after taking human capital differences into account [9, 11, 12, 12, 13, 14]. Likewise, women are overrepresented in high strain occupations and have lower levels of job control even after taking occupation and title into account [7, 12].

Self-employment may be particularly consequential for cardiovascular disease, in that it may have both direct and indirect effects on risk factors for cardiovascular disease. Directly, increased work flexibility in the context of self-employment may allow more time for engagement in health-promoting behaviors such as cooking, physical activity and accessing preventive health care [15]. Indirectly, the independence that the self-employed possess may buffer the effects of high workloads, potentially reducing work-related stress and engagement in health-damaging behaviors such as smoking and binge drinking [16]. Additionally, this flexibility may help to remediate the work-home conflict that is often experienced by women [17, 18]. Lastly, among individuals from racial and ethnic minority groups and women, self-employment may provide a buffer against actual or perceived workplace discrimination, lowering chronic stress and improving the cardiovascular disease risk profile [19, 9, 20].

One study using Health and Retirement survey data found that self-employed women, relative to women working for wages or salary reported less hypertension, obesity, diabetes and physical inactivity, adjusting for access to health care and exposure to neighborhood disadvantage [21]. Another study using Behavior Risk Factor Surveillance Survey data found that self-employed Non-Hispanic Black women in the highest income tercile were less likely to report physical inactivity, hypertension and poor physical health days, relative to their counterparts working for salary or wages.^22^]. One important limitation of these prior studies is the reliance on self-reported measures of cardiovascular disease risk factors which limits the validity of the findings. Specifically self-reported health measures have been found to be less reliable among individuals from racial and ethnic minority groups and among individuals with low socioeconomic status^23^] and self-reported weight in particular is consistently biased across all individuals [24]. Furthermore, no studies have considered the prospect of differential relationships between self-employment status and cardiovascular disease (CVD) risk factors contingent on race, ethnicity and sex. In this study, some of the shortcomings of prior studies were addressed by using National Health and Nutrition Examination Survey (NHANES) data to explore the relationship between self-employment status and cardiovascular disease risk factors across racial and ethnic minority status as well as sex. NHANES is particularly well-suited to address this research question because of the collection of objective measures of cardiovascular risk factors, inclusion of a more comprehensive set of cardiovascular risk factors and the oversampling of respondents from racial and ethnic minority groups. It was hypothesized that self-employment would be associated with a more favorable cardiovascular disease risk profile among racial and ethnic minorities and women, relative to non-minority men.

## Methods

The study was exempted from review by the UCLA Institutional Review Board. This study followed the STROBE reporting guidelines for observational studies [25].

### Study data

This study made use of NHANES data covering the years between 1999 and 2018. NHANES is a nationally representative survey of roughly 5000 individuals conducted annually by the Centers For Disease Control and Prevention to assess the health and nutritional status of adults and children [26]. NHANES is unique in combining survey and physical exam data. The survey interview covers demographic, socioeconomic, dietary, and health-related questions while the physical exam component covers medical, dental and physiologic measures as well as laboratory tests.

### Study cohort

The study population was limited to working, non-pregnant adults between the ages of 30 and 62, which captures individuals who are most likely to have completed their educational attainment and who are not yet eligible to claim social security benefits. Pregnant individuals were excluded due to the prospect of changes in health specific to pregnancy. Time for education completion was allowed to provide sufficient opportunity for individuals to start a business [27]. Limiting the sample to adults ineligible for social security benefits reduced the risk of self-selection whereby individuals who remain in the work force beyond the age of 62 are likely to be healthier than their peers [28].

Race and ethnicity are self-reported by the respondent in NHANES and then recoded to one of the following categories: Mexican American, other Hispanic, Non-Hispanic White, Non-Hispanic Black, and Other Race (includes multi-race). All individuals not classified as Non-Hispanic White were considered as belonging to a minority group for the purposes of this study. Sex is assigned in NHANES. An indicator variable for “self-employment” status was created based off of the NHANES question “Looking at the card, which of these best describes this job or work situation?”. The indicator was coded as “1” for a response of “self-employed in own business, professional practice or farm” and coded as “0” for either of the following responses: “An employee of a private company, business, or individual for wages, salary, or commission”; “a federal government employee”, “a state government employee” or “a local government employee.”

The study outcomes included several measures of CVD risk factors, coded as indicator variables reflecting well-established thresholds for enhanced CVD risk [29]. The indicator for elevated cholesterol was coded as “1” for self-reported elevated cholesterol, use of cholesterol medications or a lab value for total cholesterol ≥ 200 mg/dl. An indicator for hypertension was coded as “1” for self-reported hypertension, use of hypertension medications, a measured average systolic blood pressure of ≥ 140 mm Hg or diastolic blood pressure of ≥ 90 mm Hg. An indicator for glucose intolerance (considers pre-diabetes and diabetes) was coded as “1” for self-reported diabetes, use of diabetes medications or a hemoglobin A1c measure ≥ 5.7%. An indicator for obesity was coded as “1” for a body mass index (BMI) measure ≥ 30 kg/m^2^. An indicator for fair or poor diet was coded as “1” if the Healthy Eating Index, a validated measure of dietary quality that considers intake of fruits, vegetables, whole grains and sodium was < 50. An indicator for physical inactivity was coded as “1” if an individual reported getting less than 150 min of physical activity per week. An indicator for smoking was coded “1” if an individual reported smoking currently. An indicator for binge drinking was coded as “1” if an individual reported consuming ≥ 5 alcohol-containing beverages in a single day within the last 12 months. We created an indicator for sub-optimal sleep duration based off of the question “How much sleep do you usually get at night on weekdays or workdays?”. The indicator was coded as “1” if the response was less than seven or greater than 9 h. Lastly, we created an indicator for poor mental health that was coded as “1” if an individual reported ≥ 1 poor mental health day within the last 30 days. We adjusted our models for several potential confounders of the relationship between self-employment status and CVD risk including age, education, marital status, household poverty-to-income ratio (PIR), number of months working at current job and second-hand smoke exposure (second-hand smoke exposure was only controlled for in models of total cholesterol, hypertension and glucose intolerance) [4, 30].

### Statistical analysis

Frequencies and percentages of primary predictors, covariates and outcomes were calculated. Covariates and outcomes were also compared across self-employment status within four subgroups defined by racial and ethnic minority status as well as sex (e.g. non-minority men, minority men, non-minority women and minority women). Logistic regression models were developed to estimate the association between self-employment status and the specified CVD risk factors. These models were stratified by race, ethnicity and sex to explore differential effects across subgroups. The selection of covariates was informed by prior literature on CVD risk and self-employment, including known or suspected confounders as follows: age, education (categorized as less than high school, high school graduate/some college, and college graduate), marital status (married vs. not married), household poverty income ratio (PIR, categorized as < 200% federal poverty level, ≥ 200% federal poverty level, and unknown category), health insurance status (insured vs. uninsured) months at current job (log-transformed to address right-skewed distribution), and second-hand smoking exposure. An interaction term between self-employment status and the group variable (race, ethnicity and sex) was included to assess whether the relationship between self-employment and CVD risk varied across subgroups, prior to running stratified models. The coefficient estimates were expressed as predictive margins, that is, the change in the probability of the CVD risk factor associated with going from a value of “0” to “1” for a dichotomous predictor or change associated with a 1-unit increase in a continuous predictor [31]. A *p*-value of < 0.05 was considered statistically significant. All adjusted models were weighted for complex survey design using survey weights provided by NHANES. Missing data for outcomes was handled using complete case analysis [22], with only observations containing data for each outcome included in the models. The proportion of missing data for outcomes ranged from 0.00 to 46.1%, with higher missingness for binge drinking (17.1%), mental health status (46.1%), and poor sleep (25.9%). The extent of missing data for covariates was minimal, except for the household PIR, which had 1,516 missing values. The education variable had 10 missing values, marital status had 209 missing values and months at current job had 20 missing values, out of a total of 19,395 observations. For covariates, missing continuous values were imputed using the mean, and missing categorical values were imputed using the most frequent category. The missing PIR values were coded as “missing.” Statistical analyses were conducted between April and August of 2023. Analyses were performed using SAS 9.4 (Copyright (c) 2002–2012 by SAS Institute Inc., Cary, NC, USA).

## Results

Our initial cohort consisted of 28,518 adults aged 30–62 years from the NHANES dataset (1999–2018). After excluding non-working individuals (*n* = 8,794) and pregnant women (*n* = 329), the final sample included 19,395 participants. Of these 2104 (10.9%) were self-employed. A total of 3584 (18.5%) were non-minority women, 5332 (27.5%) were minority women, 4382 (22.6%) were non-minority men and 6097 (31.4%) were minority men. Mean age was 45 years (SD = 8.9). The proportion of self-employed individuals were 15.5%, 9.8%, 10.1% and 7.3% for non-minority men, minority men, non-minority women and minority women, respectively. A total of 6123 (31.6%) had PIR < 200%, 4368 (22.6%) were uninsured and 3998 (20.6%) had less than a high school degree. The mean number of months working at the current job was 106.

Comparisons of sociodemographic variables and CVD risk factors, across self-employment status and minority/sex subgroup are shown in Tables 1 and 2. Self-employment was associated with a higher education level among non-minority women (47.5% vs. 37.6% with graduate degree) and with a lower education level among minority women (29.5% vs. 20.7% with less than high school degree). Additionally, self-employment was associated with a higher probability of having a PIR < 200% among non-minority men (18.8% vs. 13.3%) and among minority women (49.3% vs. 35.5%).

**Table 1:** Sociodemographic variables by self-employment status stratified by Racial/ethnic minority status and sex

	Non-Minority Men			Minority Men			Non-Minority Women			Minority Women		
Self Employed	No	Yes	*p*-value	No	Yes	*p*-value	No	Yes	*p*-value	No	Yes	*p*-value
Table 1. Unadjusted comparisons of demographic factors by self-employment status stratified by racial/ethnic minority status and sex												
**Age**	45.1 (0.2)	47.3 (0.4)	< 0.001	43 (0.1)	45.3 (0.5)	< 0.001	45.7 (0.2)	46.9 (0.5)	0.03	43.8 (0.2)	45.6 (0.6)	0.003
**Months at Current Job**	113.2 (2)	174.1 (6.2)	< 0.001	84.7 (1.6)	118.2(5.4)	< 0.001	101.1 (2.1)	128.3 (6.3)	< 0.001	83.6 (1.6)	109.1 (6.8)	< 0.001
**Education**:			0.28			0.58			0.001			0.003
**Less Than High School**	8	9.6		29.3	28.8		6.3	3.6		20.7	29.5	
**High School Graduate**	54	51.8		46.9	45.1		56.1	48.8		52.5	45.2	
**College Graduate**	38	38.6		23.8	26.1		37.6	47.5		26.8	25.3	
**PIR < 200%**	13.3	18.8	< 0.001	35.1	38.6	0.26	15.1	16.8	0.56	35.5	49.3	< 0.001
**No Insurance**	10.7	25.1	< 0.001	29.2	49.5	< 0.001	8.5	17.7	< 0.001	21.1	45.6	< 0.001
**Married**	72	70.5	0.44	65.6	63.3	0.39	65.7	71.3	0.05	49.6	(57.2	0.01
**Second-hand Smoke Exposure**	15.2	18.5	0.05	13.8	12.8	0.60	15	12.7	0.19	13.7	8.9	0.03

The data source was the National Health and Nutrition Examination Survey (1999–2018). The study population included working, non-pregnant adults (ages 30–62). Bivariate analyses were conducted using “test of proportions” and T-test for dichotomous and continuous measures, respectively. Percentages were reported for dichotomous variables and means and standard errors were reported for continuous variables

**Table 2:** Unadjusted comparisons of CVD risk factors by self-employment status stratified by Racial/ethnic minority status and sex

	Non-Minority Men			Minority Men			Non-Minority Women			Minority Women		
Self Employed	No	Yes	*p*-value	No	Yes	*p*-value	No	Yes	*p*-value	No	Yes	*p*-value
**High Cholesterol**	63.2	65.8	0.22	58.8	62.3	0.19	58.1	56	0.45	50.6	55.8	0.13
**High Blood Pressure**	53	51	0.36	52.3	51.2	0.69	40.7	39	0.61	45	43.7	0.68
**Diabetes**	21.6	22.4	0.66	36.4	37.1	0.76	20.3	14.6	0.03	33.5	33.7	0.94
**Obese**	36.4	34.2	0.27	34.4	34.4	0.97	36.9	28.8	0.006	42.7	38.3	0.17
**Poor Diet**	43.1	36.5	0.005	40.6	40	0.83	37.1	31.3	0.05	37.2	30	0.03
**Physical Inactivity**	59.4	58.3	0.64	66.4	64.7	0.5	63.2	54.7	0.004	74.1	69.7	0.1
**Current Smoking**	22.6	21.6	0.59	24.1	21.9	0.3	20.7	17.4	0.12	16	12.9	0.2
**Binge Drinking**	51.4	47.3	0.09	43.8	47.2	0.25	27.3	25.3	0.53	18	17.5	0.83
**Poor Sleep**	38.1	35.4	0.33	44.9	40.2	0.1	31	20.7	0.003	43.8	35.3	0.02
**Poor Mental Health**	34.4	36.9	0.36	29.7	30.4	0.79	49.1	40.7	0.02	42.6	46.3	0.33

The data source was the National Health and Nutrition Examination Survey (1999–2018). The study population included working, non-pregnant adults (ages 30–62). Bivariate analyses were conducted using “test of proportions”. Percentages were reported for dichotomous variables

CVD = Cardiovascular Disease

With respect to the unadjusted comparisons of CVD risk factors across self-employment status, among non-minority women, self-employment was negatively associated with glucose intolerance (14.6% vs. 20.3%, *p* = 0.033), physical inactivity (54.7% vs. 63.2%, *p* = 0.004), obesity (28.8% vs. 36.9%, *p* = 0.006), poor mental health (40.7% vs. 49.1%, *p* = 0.021) and poor sleep (20.7% vs. 31%, *p* = 0.003). Among minority women, self-employment was negatively associated with poor diet (30% vs. 37.2%, *p* = 0.028) and poor sleep (35.3% vs. 43.8%, *p* = 0.019) in the unadjusted analyses. Among non-minority men, self-employment was negatively associated with poor diet before adjustment (36.5% vs. 43.1%, *p* = 0.005). We did not find any significant associations between self-employment and CVD risk factors among minority men in unadjusted analyses.

Adjusted comparisons of CVD risk factors and self-employment status are shown in Table 3. Among non-minority women, the negative associations between self-employment, glucose intolerance, (13% vs. 19.2%; % diff = -6.2%; 95% CI: -10.6%, -1.8%; *p* = 0.005), physical inactivity (63.7% vs. 70.7%; % diff = -7.0%; 95% CI: -12.7%, -1.3%; *p* = 0.017), obesity (27.6% vs. 34.9%; % diff = -7.4%; 95% CI: -12.8%, -1.9%; *p* = 0.008) and poor sleep (21.8% vs. 31.2%; % diff = -9.4%; 95% CI: -15.7%, -3.0%; *p* = 0.004), remained statistically significant post adjustment, whereas differences in poor mental health were no longer statistically significant. Among minority women, negative associations between self-employment, poor diet (27.9% vs. 34.6%; % diff = -6.7%; 95% CI: -12.5%, -0.9%; *p* = 0.024) and poor sleep (34.4% vs. 42.6%; % diff = -8.1%; 95% CI: -14.8%, -1.5%; *p* = 0.017) remained statistically significant. Additionally, self-employment became negatively associated with being physically inactive after adjusting for covariates (69.9% vs. 77.2%; % diff =-7.3%; 95% CI: -13.1%, -1.5%; *p* = 0.013) among minority women. Among non-minority men, self-employment remained negatively associated with poor diet (38.1% vs. 44.6%; % diff = -6.5%, -1.7%; 95% CI: -11.3%,; *p* = 0.008) after adjustment. Furthermore, self-employment became negatively associated with hypertension (47% vs. 52.7%; % diff = -5.7%; 95% CI: -10.1%, -1.2%; *p* = 0.013) in the adjusted analysis. Lastly, we did not find any significant associations between self-employment and CVD risk factors among minority men in adjusted analyses.

**Table 3:** Adjusted comparisons of CVD risk factors by self-employment status stratified by Racial/ethnic minority status and sex

% Difference 95% CI *p*-value	Non-Minority Men	Minority Men	Non-Minority Women	Minority Women
**High Cholesterol**	-0.5%	0.3%	-3.80%	2.90%
	(-4.9%, 3.9%)	(-5.2%, 5.7%)	(-9.6%, 1.9%)	(-3.8%, 9.7%)
	*p* = 0.83	*p* = 0.92	*p* = 0.19	*p* = 0.40
**High Blood Pressure**	-5.70%	-4.60%	-2.10%	3.60%
	(-10.1%, -1.2%)	(-10.5%, 1.3%)	(-8.8%, 4.5%)	(-10.1%, 2.9%)
	*p* = 0.01	*p* = 0.13	*p* = 0.54	*p* = 0.28
**Diabetes**	-2.30%	-4.00%	-6.20%	-3.80%
	(-5.8%, 1.2%)	(-8.9%, 0.9%)	(-10.6%, -1.8%)	(-9.5%, 1.9%)
	*p* = 0.19	*p* = 0.11	*p* = 0.005	*p* = 0.19
**Obesity**	-3.00%	-0.70%	-7.40%	-4.80%
	(-6.9%, 0.8%)	(-5.1%, 3.8%)	(-12.8%, -1.9%)	(-10.9%, 1.2%)
	*p* = 0.12	*p* = 0.78	*p* = 0.008	*p* = 0.12
**Poor Diet**	-6.50%	0.20%	-4.10%	-6.70%
	(-11.3%, -1.7%)	(-5.3%, 5.7%)	(-10.1%, 1.9%)	(-12.5%, -0.9%)
	*p* = 0.008	*p* = 0.94	*p* = 0.18	*p* = 0.02
**Physically inactivity**	-2.40%	-2.50%	-7.00%	-7.30%
	(-6.9%, 2.1%)	(-7.5%, 2.6%)	(-12.7%, -1.3%)	(-13.1%, -1.5%)
	*p* = 0.29	*p* = 0.34	*p* = 0.02	*p* = 0.01
**Current Smoking**	0.50%	-0.70%	-0.20%	-2.60%
	(-4.1%, 5.1%)	(-4.9%, 3.5%)	(-5.3%, 5.0%)	(-6.3%, 1.0%)
	*p* = 0.83	*p* = 0.74	*p* = 0.95	*p* = 0.16
**Binge Drinking**	-1.60%	6.20%	-0.10%	1.60%
	(-6.3%, 3.1%)	(0.0%, 12.5%)	(-6.6%, 6.3%)	(-3.6%, 6.7%)
	*p* = 0.50	*p* = 0.05	*p* = 0.97	*p* = 0.55
**Poor Sleep**	-3.90%	-5.40%	-9.40%	-8.10%
	(-9.5%, 1.7%)	(-10.9%, 0.2%)	(-15.7%, -3.0%)	(-14.8%, -1.5%)
	*p* = 0.17	*p* = 0.06	*p* = 0.004	*p* = 0.02
**Poor Mental Health**	3.80%	1.70%	-7.20%	4.50%
	(-1.9%, 9.4%)	(-3.6%, 7.1%)	(-14.7%, 0.4%)	(-3.4%, 12.4%)
	*p* = 0.20	*p* = 0.53	*p* = 0.06	*p* = 0.26

The data source was the National Health and Nutrition Examination Survey (1999–2018). The study population included working, non-pregnant adults (ages 30–62). Logistic regression models were used to estimate the association between self-employment status and Cardiovascular Disease (CVD) risk factors. The point estimates were given as predictive margins, the percentage point change in the CVD risk factor associated with self-employment, relative to working for wages or salary

## Discussion

This cross-sectional analysis explored the relationship between self-employment and CVD risk factors across subgroups defined by racial/ethnic minority status and sex. This study demonstrated that non-minority women, followed by minority women had the most favorable CVD risk profile associated with self-employment. Non-minority men had less hypertension and reported a lower risk of fair or poor dietary quality associated with self-employment. Among minority men, no relationships with CVD risk factors were observed. These results are largely supportive of the study hypothesis with the exception that self-employment was not associated with a favorable CVD risk profile among minority men. Nonetheless, this study builds on prior work on this topic by using more reliable and valid measures of CVD risk factors (e.g. cholesterol, hypertension, glucose intolerance, dietary quality) and adjusting results for a more comprehensive set of potential confounders (household poverty-to-income ratio, number of months working at current job and second-hand smoke exposure) [32, 33]. Furthermore, this study included an additional measure of CVD risk, relative to other studies on this topic (sleep duration). Lastly, no studies have previously explored the relationship between self-employment status and CVD risk across sex along with racial and ethnic minority status.

The results of this work are largely consistent with the results Dzodzomenyo et al. in finding a negative association between self-employment, obesity, diabetes and physical inactivity among women [21]. Likewise, Narain et al., demonstrated similar findings among Non-Hispanic Black women with respect to physical activity (all subgroups of women) and dietary quality (subgroups comprised of the bottom two income terciles) [22]. However, unlike Dzodzomenyo et al. and Narain et al. this study did not find a negative association between self-employment and hypertension among women. This discrepancy may reflect differences in the study populations and/or differences in the prevalence of undiagnosed hypertension across study cohorts.

The lack of an association with self-employment and CVD risk factors among racial and ethnic minority men may reflect a different self-employment context for this subgroup. Minority men who are self-employed may be concentrated in business sectors with high barriers to entry and high failure rates [34]. Furthermore, businesses of minority men may struggle under the weight of lower financial capital and reduced access to mentorship [34]. As such, the stress of maintaining a business under these circumstances may counter the potential health benefits of work flexibility and autonomy [35].

The association of self-employment with CVD risk factors among women observed in this study is not trivial. Poor dietary quality is the leading risk factor implicated in mortality from noncommunicable disease, surpassing tobacco in recent years [36]. Physical activity has been linked to improvements in cardiovascular and all-cause mortality, irrespective of BMI [37]. Furthermore, short sleep duration has been linked with obesity, stroke risk and Hypertensive Disorders of Pregnancy [3, 38, 39]. Lastly, excess weight and glucose intolerance are among the top 5 risk factors implicated in the high burden of chronic disease in the United States [36]. That said, work structure may be a clue for helping to illuminate cardiovascular disease risk. Screening for work context may ultimately prove to be an additional strategy for identifying patients that might be at high risk for adverse cardiovascular outcomes and consequently ideal targets for more aggressive screening. Furthermore, work context is heavily correlated with race, ethnicity and sex [10] and may be a vehicle for perpetuating disparities in cardiovascular disease prevalence, morbidity and mortality [4]. Specifically, research has shown that compared to white individuals, people of color earn less, are evaluated as less knowledgeable and less effective, have fewer promotion prospects, have less job security, and report lower levels of job satisfaction [11]. This study starts to lay the foundation for future studies that may shed light on how the psychosocial experience of work contributes to higher cardiovascular disease risk for many people of color and subsequently strategies to remediate this risk.

### Study limitations

The results of this study must be interpreted in the context of several limitations. As this is a cross-sectional analysis, no causal claims can be made. Particularly, reverse causality cannot be ruled out as an explanation for these findings. Individuals with chronic diseases, such as hypertension, may be less likely to choose self-employment due to the desire to maintain employer-sponsored health insurance benefits [40]. Additionally, there may be unmeasured characteristics that simultaneously impact the propensity to choose self-employment and CVD risk factors (e.g., personality traits and coping strategies) [41]. Furthermore, we were unable to distinguish between individuals who chose self-employment out of business interest (“opportunity” self-employment) and individuals who may have been pushed into self-employment due to unemployment, or other unfavorable circumstances (“necessity” self-employment) [42, 43]. Studies suggest that the relationship between self-employment and health varies across opportunity vs. necessity self-employment status [44]. We attempt to minimize this limitation by controlling for poverty-income-ratio which may serve as a proxy for self-employment type given that opportunity entrepreneurs will have higher earnings than necessity entrepreneurs on average. We also cannot guarantee that individuals who report self-employment are not also engaged in work for wages. This is particularly relevant for women from racial and ethnic minority groups who often start businesses while maintaining work for wages [45]. This limitation could bias the findings for minority women towards the null. Lastly, sample size limitations preclude further stratification of the data along racial and ethnic lines. Consequently, the relationship between self-employment and CVD risk factors may vary across racial and ethnic groups categorized as minority.

Despite these study limitations, there may be important lessons to glean from these results.

It is unrealistic to expect that all women will become self-employed; however, it may be worth considering how some of the positive features of self-employment such as increased autonomy and flexibility may be imported into the wage-employment context. Flex-time (a work arrangement that allows employees to choose the start and end time for their workday) is strategy used by some employers to promote employee autonomy and allows employees some measure of control in their work schedule [46]. Flex-time is generally viewed favorably by employees and has been associated with increased motivation, productivity and job satisfaction [46]. Furthermore, Berkman et al. (2023) found that information technology and long-term care workers with high baseline cardiometabolic risk randomized to an intervention to increase workplace flexibility and supervisor support and decrease work–family conflict had a reduction in cardiometabolic risk on par with a 5-to-10-year reduction in age-related changes [47]. Consequently, implementing well-structured flex-time policies could result in improvements in women’s health. Future studies that incorporate methods capable of identifying a causal relationship between self-employment and CVD risk among women are needed.

## Conclusion

We conducted a cross-sectional analysis exploring the relationship between self-employment and CVD risk factors across subgroups defined by racial and ethnic minority status as well as sex. We found that self-employment was associated with a favorable CVD risk profile among women, particularly non-minority women. The relationship between self-employment and CVD risk factors among men was more muted and varied across minority status. This study suggest that work context may provide clues for CVD risk; however, this relationship may differ along racial, ethnic and sex lines. Furthermore, additional research is needed to further characterize this relationship.

## Data Availability

The datasets used and/or analysed during the current study available from the corresponding author on reasonable request.

## Abbreviations

CVD: Cardiovascular Disease
NHANES: National Health and Nutrition Examination Survey
PIR: Poverty-to-income ratio
